# Ethnobotany of traditional medicinal plants and associated indigenous knowledge in Dawuro Zone of Southwestern Ethiopia

**DOI:** 10.1186/s13002-022-00546-4

**Published:** 2022-06-21

**Authors:** Mathewos Agize, Zemede Asfaw, Sileshi Nemomissa, Tizazu Gebre

**Affiliations:** 1 Department of Biology, College of Natural Sciences, Arba Minch University, P.O. Box 07, Arba Minch, Ethiopia; 2 Department of Biology, College of Natural and Computational Sciences, Wolaita Sodo University, P.O. Box 138, Wolaita Sodo, Ethiopia; 3 Department of Plant Biology and Biodiversity Management, College of Natural Sciences, Addis Ababa University, P.O. Box 3434, Addis Ababa, Ethiopia

**Keywords:** Dawuro, Ethnobotany, Medicinal plants, Traditional herbal medicine knowledge

## Abstract

**Background:**

The study aimed at documenting the indigenous and local knowledge and use of traditional medicinal plants for treating human and livestock ailments in Dawuro Zone of Ethiopia.

**Methods:**

A survey was conducted among traditional healers and native administrators through discussion, interviews, and field observations. The snowball sampling technique was used to select 384 traditional healers in purposefully selected 50 villages spanning seven districts for face-to-face individual interviews. The chi-square test was applied to establish associations between traditional healers’ demographics, the distance between the village site and the nearest natural forest and a health center, and SPSS V.20 software was used for the analysis.

**Results:**

The traditional healers of the study area reported the use of 274 traditional medicinal plant species belonging to 217 genera and 82 families. Asteraceae (11.68%), Fabaceae (9.49%), and Lamiaceae (9.12%) were the foremost frequently used families. Herb species (54.8%) and leaves (65%) were predominantly sourced from the wild environment. The quantity of medicinal plants used (*x*^2^ = 278.368, *df* = 20, *P* = 0.000) and years of (experience in) traditional healing using herbs (*x*^2^ = 76.358, *df* = 10, *P* = 0.000) varied with distance from the natural forests. The service charge for healing had strong positive association (*x*^2^ = 24.349, *df* = 5, *P* = 0.000) with healer’s age (*x*^2^ = 309.119, *df* = 184, *P* = 0.000) and educational level (*x*^2^ = 851.230, *df* = 598, *P* = 0.000) with distance of traditional healer's residence from the medical institution. The agricultural activities, urbanization, low or no charge for the healing service, the secrecy and oral transfer of the knowledge, and the demand for medicinal and other multiple purposes species were some of the factors threatening the resource and the associated knowledge as well as the service in the study area.

**Conclusion:**

There are diversified traditional medicinal plants applied for healthcare of the community and domestic animals of the study area. The source of remedies mostly depends on herbs of natural forests, and the leaf was the most frequently used plant part. Developing conservation intervention and sustainable systems of utilization is needed for multipurpose medicinal plants. Finally, integrating with modern system and formalizing, legalizing, and capacitating the traditional medicine practitioners are needed for access of primary healthcare systems to rural communities.

**Supplementary Information:**

The online version contains supplementary material available at 10.1186/s13002-022-00546-4.

## Background

Documenting community knowledge from older adults requires urgent and multidisciplinary engagement before knowledgeable people die. Ethnobotanical, ethnomedicinal, and anthropological researchers must continue researching herbal medicine so as to know the cultural, sociological, and practical considerations that inform the broader community at institutional and governmental level [1]. Herbal treatments were used before modern drugs [2]; hence, scientists and pharmaceutical companies are targeting to find new drug sources from traditional medicine [3]. People of different localities in the world possess peculiar knowledge of plant resources on use for medicine and other [4, 5]. Currently, over 80% of the world’s population [6] and in the developing countries including Africans [6–9] relies on traditional healing practice and traditional medicinal plants **(**TMPs) to tackle ailments. In rural areas of Ethiopia, about 70% human and 90% livestock population depend upon traditional medicine (TM) [10]. The utilization of TM in Ethiopia is estimated between 60 and 79% with 600 herbal medicine practitioners [11]. The traditional healthcare is culturally deep-rooted without documenting and practical mock-up.

Traditional medicine is the practice handed down through generations [12]. Drug discovery from plants today is a fashionable and lengthy process [13]. Its global market size has increased in billions [14]. Hence, traditional medicines have emerged as an alternate treatment of chronic diseases and lifestyle disorders [9, 13]. Most traditional medicinal preparations are of plant origin [6, 9, 14] to treat disease and enhance general health and well-being [15, 16]. Traditional medicine plays a big role, and therefore the overwhelming majority of the population lives in rural areas with little access to modern health services [17, 18].

Traditional healers were the first line of healthcare providers in the community. Almost all/most of modern healthcare systems were derived from the traditional system through modernization of the collection, preparation, storage, use, application, and delivery of the service. The documentation of indigenous knowledge counted centuries and is still ongoing in a fragmented manner. Worldwide policies, regulations, and guidelines have been developed for encouraging the use of traditional medicinal practices, but this is not commensurate with its use and the extent of attention given to the modern healthcare system. Interventions are needed for formulating workable policies for collaboration and the evaluation of diseases by modifying health intervention programs to suit local conditions [19]. There was a boost of governmental organizations and non-governmental organizations like WHO established to ensure it. In developing countries, due to lack of budget, there was no training directed to the knowledgeable people [20]. The training could capacitate them to the extent of registering service provision and integrating with the modern primary healthcare system at grassroots level [20]. Also, a budget allocation is needed in order to carry out nationwide assessment before knowledgeable elder persons die.

In India, traditional healthcare systems are relevant and aid within the treatment of chronic illnesses; China and Japan are on the forefront considering the mixing of traditional medicine into the primary healthcare system [21]. Within the African context, in Kenya, traditional medicine practitioners do not disclose to patients about their practice [21]. But in the study area, the traditional healing system is as yet not given legal ground. If traditional healers are trained and get integrated with the modern healthcare system, the community would be better served. They have time-honored knowledge about the use and management of the traditional medicinal plants. They serve occasionally and that they do not consider it as their business because their job is farming. Due to this, there could be leaking of information about the healthcare system among the community. The study used the distance from the homes of herbalists to the nearby natural forest, health center, and urban area to test the effect on service delivery as hypotheses. More use of traditional herbal medicine when distance between the healer’s home is closer to the natural forest, further from the health center and from urban center. This study was dispensed to document traditional healing systems, traditional medicinal plants used and ailments treated and also the way they perpetuate their practice to fill the prevailing gaps of the healthcare system in Dawuro Zone of southwestern Ethiopia.

## Materials and methods

### Description of the study area

**Fig. 1:**
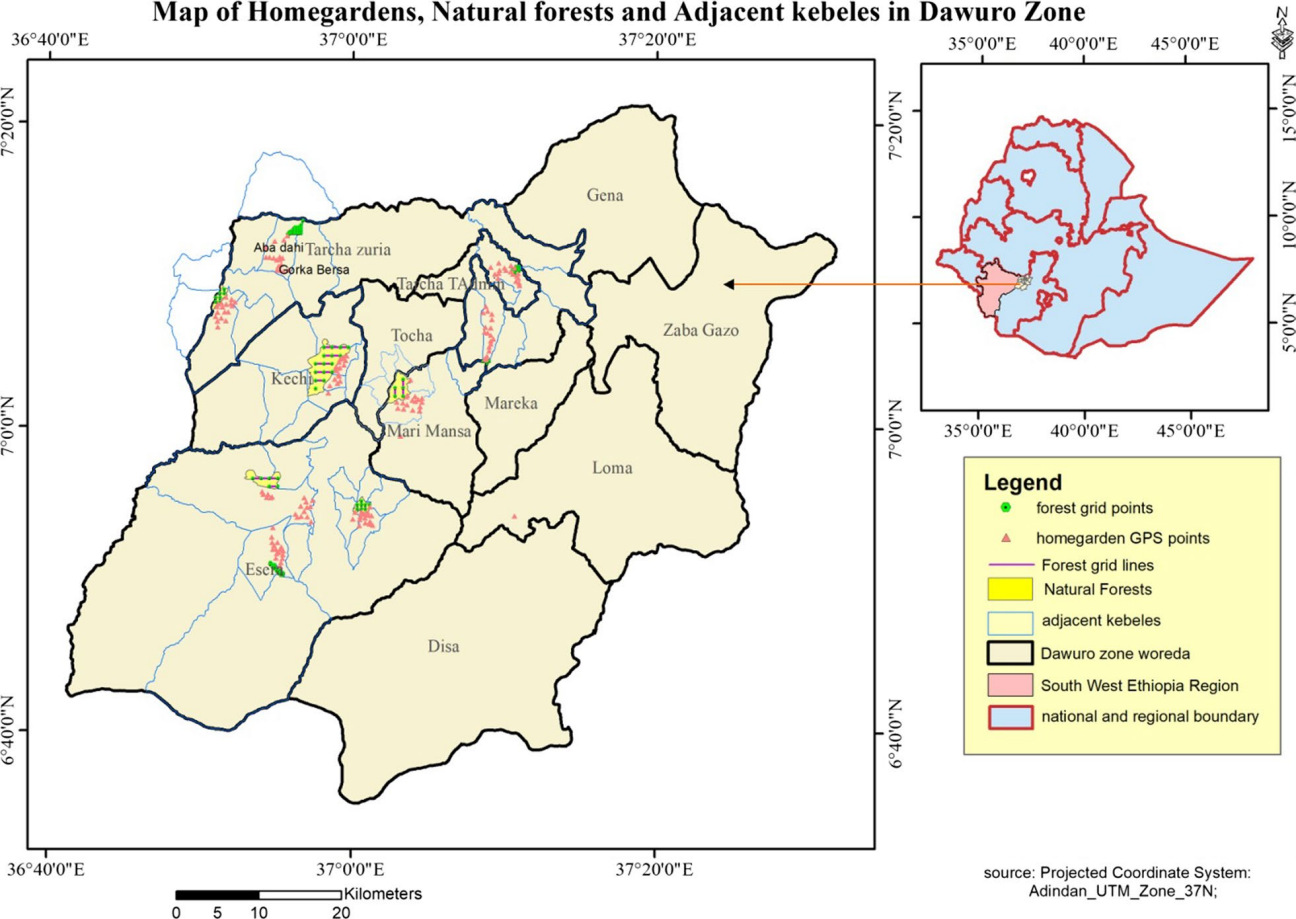
Map of Ethiopia showing the location of the study area with the total and the sampled districts

Dawuro zone is one among the six zones within the southwestern region of Ethiopia. Dawuro lies in between 6° 36′ to 7° 21′ north latitudes and 36° 68′ to 37° 52′ east longitudes [22]. The population of Dawuro was estimated to be 600,121 and density of 135.28/km^2^ [23]. Currently, there are 10 districts and two town administrations within the zone, namely Maraka, Tocha, Esara, Kechi, Tarcha Zuria, Mari-Mansa, Loma Bosa, Disa, Gena and Zaba Gazo districts, and Tarcha and Gasa Town Administrations [24]. Of these, the first six districts and Tarcha Town Administration were selected purposely (Fig. 1) as data collection sites. The districts are with highly undulating and scrutinized surfaces posing extreme difficulties for undertaking ethnobotanical surveys. The peak point of the study area is 2820 m above sea level (masl), and the lowest is 501 masl [23].

The study area has three agroecological areas, namely Dega (highland or land > 2300 masl), Woina Dega (midland or 1500–2300 masl), and Kolla (lowland or < 1500 masl) [22]. The annual average maximum and minimum temperature is 29 °C and 16 °C, respectively, with annual rainfall of 900–1620.3 mm [23]. The study area gets rainfall almost throughout the year, and also the amount is high during the northern summer due to winds coming from the Gulf of Guinea [23]. About 85% of the people of the study area rely on agriculture, and therefore the other 15% on different jobs including government workers or professionals. The vegetation varies from *Combretum-Terminalia* types to broad-leaved, deciduous woodland in lowland areas, dry evergreen montane forest and grassland complex in midland and moist evergreen forest, broad-leaved evergreen forest types within the highlands [20]. The forest cover of Dawuro Zone is about 137,308.825 ha, which is 30% cover of the land and of this, the study area forest covers about 53,201.575 ha [22]. *Ensete ventricosum* (the Ethiopian banana-like root/tuber crop), which provides vegetation cover and creates green scenery, is one of the staple foods within the area.

### Data collection methods

#### Determination of sample size and sampling techniques

In case of determination of sample size and sampling techniques, six districts and one town administration were selected purposefully. The Kebele (subdistrict) numbers were decided by employing the quota sampling method. Sample size for each Kebele was calculated using the proportion of the number of households in each Kebele to the full number of households of each district. The specified sample size of the traditional herbal medicine practitioners was selected by using snowball sampling technique (to merit selection at least three to five individuals should provide information about a knowledgeable practitioner, who is a man or a woman/him or her in our case), but their number was determined by using standard sample size determination formula [25] assuming *P* = 0.5 (maximum variability), by a 95% confidence level and ± 5% precision. $No = \frac{{Z^{2} pq}}{{e^{2} }} = \frac{{\left( {1.96} \right)^{2} \left( {0.5} \right)\left( {0.5} \right)}}{{\left( {0.05} \right)^{2} }} = 384$ which is valid where “No” is the sample size, “*Z*^2^” is the abscissa of the normal curve that cuts off an area “*a*” at the tails (1 − *a* equals the desired confidence level, e.g., 95%) “1,” “*e*” is the desired level of precision, “*p*” is the estimated proportion of an attribute that is present in the population, and “*q*” is 1 − *p*. The value for *Z* is found in statistical tables which contain the area under the normal curve.

#### Ethnobotanical field data collection

Semi-structured interviews, focus group discussions, participant observation, and walk-in-the-homegardens and natural forest patches were the methods used to collect ethnobotanical data as described by [4, 26, 27]. The five known markets were selected purposefully from the study area and visited three times. Randomly selected vendors in the market were posed questions to document the traditional medicinal plant species, their part used, form available, the reason sold and their source.

#### Plant sample collection

Plant sample collection was absolutely done with responsibility for the accomplishment of the research in keeping with [28] guidelines Version 1:4b from August 2020 to March 2021. Botanical identification of the plants was undertaken using Ethiopian Flora books from volumes 1–8 and by comparison with authenticated voucher specimens at the National Herbarium of Ethiopia (Addis Ababa University) and further confirmed by a senior taxonomic expert there (Prof. Sileshi Nemomissa). Finally, the identified triplet sample voucher specimens with their numbers and labels were deposited there at the National Herbarium of Ethiopia, the Herbarium of Arba Minch University, and the Mini-Herbarium of Dawuro Historical and Cultural Museum (Additional file 1: Appendix 1).

### Data analysis methods

Use categories and preferences were analyzed using data matrix preference or priority ranking, direct matrix ranking; informant consensus factor (ICF), fidelity level (FL), and use value. Preference ranking and direct matrix ranking were computed using the values or scores given by 12 randomly selected traditional healers as key informants on use preference and/or use diversity of medicinal plants. For the former, the scores were added and ranked and the overall degree of preference is given in rank order following [4]. For direct matrix ranking, the rank values were converted into scores with the most preferred medicinal plant that ranked first, getting the best value and the others down the line following [4]. For this, 10 top multipurpose traditional medicinal plants having high use values were selected. Participants (10 randomly selected traditional healers as key informants) were asked to assign values to different uses of each plant indicating the degree of uses (with 5 = best; 4 = very good; 3 = good; 2 = less used; 1 = least used and; 0 = no value). By adding the scores, the factors contributing to overharvesting of the medicinal plant species were identified as recommended by [27].

The informant consensus factor (ICF) was computed to work out the foremost important human and livestock ailment categories and identify potentially effective medicinal plant species within the respective disease categories. Accordingly, 274 traditional remedies and corresponding to 121 human ailments were grouped into 14 ailment categories. ICF was then obtained by using the formula: ${\text{ICF}} = \frac{{{\text{nur}} - {\text{nt}}}}{{{\text{nur}} - 1}}$ [28, 29] where nur = number of use citations in each disease category; nt = the amount of times a species was used for that cluster.

Fidelity level (FL) is computed in order to spot the most preferred traditional medicinal plant species employed by the local people against human and livestock ailments using the fidelity level (FL) index given in [30, 31] as ${\text{FL}} = \frac{{{\text{Ip}}}}{{{\text{Iu}}}} \times 100$ where Ip = the quantity of informants who independently cited the importance of a species for treating a selected disease and Iu = the whole number of informants who reported the plant for any given disease.

The use value (*UV*) of the traditionally claimed useful plant species was computed following [29] with the formula: $Uv = \frac{U}{{{\text{ns}}}}$ where “*U*” refers to the amount of uses mentioned by the informants for a given species and “ns” refers to the full number of informants interviewed.

Relative importance (RI) was computed to measure the diversity of use of plants both medicinal application and others following [32] the formula: RI = NUC + NT.

Here, NUC = number of use categories of a given species; NT = number of types of uses. NUC = NUCS/ NUCVS**);** NUCS is the number of use categories of a given species; NUCVS is the total number of use categories of the most versatile species; NT = NTS/ NTMIT **(**NTS is the number of types of uses attributed to a given species; NTMIT is the total number of types of uses attributed to the most important taxon).

### Use diversity (UD)

Use diversity values are employed to measures the use categories of a species and to see how evenly these contribute to the total uses of the species. Use diversity (UD) values of species were computed using Shannon–Wiener diversity index as given in various sources [33–35] and calculated with the formula UDs = − ∑pc*In (pc).

Where, UDs= use diversity value of species s; pc (is the contribution of use category *c* to total utility of a species *s*) is (n/N) (i.e.) the proportion of the number of times species s was mentioned by all participants within each category (n) divided by the total number of use reports of species s across all use categories (N), ln is the natural log, Σ is the sum of the calculations, and s is the number of species.

### The cultural value (CV_***e***_)

The cultural value (CV_*e*_) of a species was computed by following [36, 37] formula, CV_*e*_ = Uc_*e*_ * Ic * IUc, where CV_*e*_ is the cultural value of species *e,* Uc_*e*_ is the total number of uses reported for species *e* divided by the total number of potential uses (e.g., food, fodder, construction), Ic is the number of participants who listed species *e* as useful divided by the total number of people participating in free listing, and IUc is the number of participants who mentioned each use of species *e* divided by the total number of participants.

The chi-square test is used to test and establish associations between THs’ demographics and the distances from natural forests and health centers. One-way analysis of variance (ANOVA) was used to compare traditional healing service fee between different age-groups, education levels, sex, family size, and therefore the resident’s distance from natural forests. Binary logistic and multiple correlation models were accustomed to identify socio-demographic predictors of respondents’ experiences, and SPSS V.20 software was used for the analysis of the resulting data.

### Ethical consideration

The study proposal was reviewed and approved by the Arba Minch University ethical clearance committee following which the university wrote a supporting letter in accordance with the national guidelines to the study area administration office. Permissions were obtained from the zone, district, and Kebele administrators to carry out the fieldwork. The data collection was made in line with the World Medical Association Declaration of Helsinki Ethical Principles [38] and in accordance with the Access and Benefit Sharing Regulation of Ethiopia [39]. Only volunteers and informed participants were involved in interviews to collect indigenous knowledge (non-clinical sample study) on herbal medicine and its application. Verbal consent was obtained before interviewing each farmer and/or traditional herbalist.

## Results

### Diversity of medicinal plants within the study area

The study found that 274 TMP species distributed in 217 genera and 82 families and used by the community of the study area occur in different forest patches and other areas. THs of the study area use Asteraceae (11.68%), Fabaceae (9.49%), Lamiaceae (9.12%), Poaceae (6.2%), Euphorbiaceae (4.02), and Solanaceae (3.65%) and also the rest 76 families cover 55.84%. The TMPs with different growth forms including 150 (54.75%) herbs, 52 (18.98%) shrubs, 50 (18.25%) trees, and also the rest covering 22 (8.02%) including 2 (0.73%) creepers, 19 (6.93) climbers, and 1 (0.36) liana were used for human and livestock remedy preparation (Additional file 1: scientific name, family name, local (Dawuro) name, habit, part used, disease treated, disease cluster, treatment for, preparation, route of administration, source, distribution, use reports).

Most of the leaf parts of 178 (65%) of the TMPs were used for ailments and also the root comes next top part used which comprises 31 species (11.31%). Of the others, fruits cover 16 (5.84%), seeds 15 (5.47%), rhizome and bulb 4 (1.46%), bark 3 (1.09%), stem 3 (1.09%), whole plant 2 (0.72%), and sap 2 (0.72%), and utilization of various parts together comprises 20 species (7.3%).

Of the total species, 117 (42.70%) of the TMPs were employed in making decoctions, 62 (22.63%) concoctions, and 95 (34.67%) were utilized in alternative ways (in either way). They were rolled with food, otherwise chewed, smoked, or applied as ointment or drop.

### Ethnobotanical knowledge of traditional medicinal plants practitioners

#### Acquisition of traditional medicinal knowledge

Acquisition of traditional medicinal knowledge happens in multiple ways. Most of (294, 76.56%) the THs said they acquired traditional healing knowledge through their family routes orally with great secrecy usually at adulthood. The remaining (90, 23.44%) healers confirmed acquiring their healing knowledge from different sources through cultural transmission, education, friends, observation, payment, reading religious books, and trial-and-error learning. About 55% of the THs who claimed to have accumulated their traditional healing practice through family lines have (${x}^{2}$=67.0, *df* = 8, *P* < 0.001) not attended school grades.

TMPs used and the ailments treated: The traditional healing practice fills the gap created by the healthcare delivery of the modern system at the grassroots level in addition to fulfilling its unique cultural and social roles. A total of 121 types of human and livestock ailments were recorded, where 70 (57.85%) of these are human health problems. These diseases were grouped into 14 clusters as accessory problems (0.803), blood vascular system problems (0.977), gastrointestinal system problems (0.703), internal problems (0.703), metabolic disorders (0.972), microbial infections (0.738), nutritional deficiency (0.939), others (0.724) including parasitic (0.902), reproductive (0.948), respiratory (0.896), skeletal (908), skin (0.695), and urinary (0.984). The results of the informant consensus factor (ICF) showed that there have been lots of TMPs commonly known to treat diseases related to urinary systems (0.984), circulatory systems (0.977), and metabolic disorders (0.972) (Additional file 2). The results of fidelity level (FL) showed that THs use plenty of plants to treat ailments. They were practiced using one to 65 TMPs. About 340 (88.54%) used from 1 to 10 TMPs, 30 (7.81%) used from 11 to 20 TMPs, but the remaining 14 (3.65%) used from 21 to 65 TMPs. Medicinal plant knowledge is positively correlated (*r* = 0.571, *α* = 0.05, *P* = 0.000) with the quantity of diseases treated. About 361 (94.01%) TH have 1–10 different knowledge of treating diseases, while the remainder 23 (5.99%) have 11–31 different diseases. It was absolutely recorded that several traditional plants are used for treating ailments of the identical ill health because it was shown in fidelity level (FL) (Table 1). Ethnobotanical knowledge on *Maerua oblongifolia* (100) and *Syzygium guineense* (99.39) had high consensus as revealed by the high FL index of medicinal plants whose roots are known to treat diarrhea.

**Table 1:** Most frequently used plants for diarrhea based on highest FL (%) (total informants = 384)

Scientific name	UR	NDT	NC	OU	IC	SRD	FL	R
*Maerua oblongifolia*	290	17	7	6	75.52	290	100	1
*Syzygium guineense*	332	13	8	20	86.46	332	99.39	2
*Ensete ventricosum*	216	10	8	10	56.25	210	97.22	3
*Coffea arabica*	123	7	7	5	32.03	115	93.49	4
*Pentas schimperiana*	322	11	8	7	83.85	300	93.16	5
*Datura stramonium*	212	7	5	2	55.21	190	89.62	6
*Artemisia afra*	126	7	6	3	32.81	110	87.3	7
*Arundinaria alpina*	144	6	5	8	37.5	100	69.44	8
*Amaranthus caudatus*	78	13	7	2	20.31	50	64.1	9
*Commelina benghalensis*	210	4	3	4	54.69	40	19.04	10

Where FL = fidelity level, IC = informants consensus, NC = number of clusters, NDT = number of diseases treated, OU = other uses, R = rank, SN = serial number, SRD = specific report of diarrhea, UR = use report

**Simple preference ranking**: The summary of the result of a simple preference ranking of 12 key informants for ten medicinal plants against liver problem also indicated the presence of potential alternative ailments to treat certain health problems having *Maerua oblongifolia*, *Syzygium guineense subsp. guineense*, and *Entada abyssinica* as the top in this order (Table 2; Additional file 1).

**Table 2:** Results of a simple preference ranking for ten medicinal plants against liver problem

Medicinal plants reported	The 12 key informants (A to L)												Total score	Rank
	A	B	C	D	E	F	G	H	I	J	K	L		
*Maerua oblongifolia*	5	5	5	5	5	5	5	5	5	5	5	5	60	1
*Syzygium guineense*	5	4	5	5	5	5	5	5	5	5	5	5	59	2
*Entada abyssinica*	5	5	4	5	5	5	5	3	5	5	5	5	57	3
*Ensete ventricosum*	5	4	5	4	4	5	5	4	5	5	5	5	56	4
*Maesa lanceolata*	4	5	4	5	4	5	4	5	4	5	5	4	54	5
*Salvia nilotica*	5	4	5	3	4	5	4	4	3	4	5	5	51	6
*Amaranthus caudatus*	4	5	3	4	5	5	2	5	3	4	5	3	48	7
*Abrus precatorius*	5	4	4	5	3	4	5	4	4	3	2	4	47	8
*Clematis hirsuta*	3	4	5	3	4	2	4	2	5	5	4	5	46	9
*Momordica foetida*	3	2	3	4	3	4	4	5	5	3	4	5	45	10
Total	44	42	43	43	42	45	43	42	44	44	45	46		

5 = the foremost preferred, but 1 is the least preferred; A to L key informants

#### The TMPs used and their sources

The informants indicated 274 traditional medicinal plant species used for treating ailments that lead to the sufferings of the local people. Each species is used to treat from one to twenty different ailments of human and domestic animals and with two to eighteen other multiple uses (Additional file 1). Most of the informants (205, 53.39%) practiced traditional herbal medicine for 11–30 years staying within the study area. Staying in a given area results in positive relationship with age and practice (*r* = 0.733, *P* = 0.000 and *r* = 0.544, *P* = 0.000, respectively) at *P* = 0.01 level, and traditional healers with knowledge of enormous number of TMPs have positive correlation statistics (*r* = 0.111, *α* = 0.05, *P* = 0.029) with family size.

**Fig. 2:**
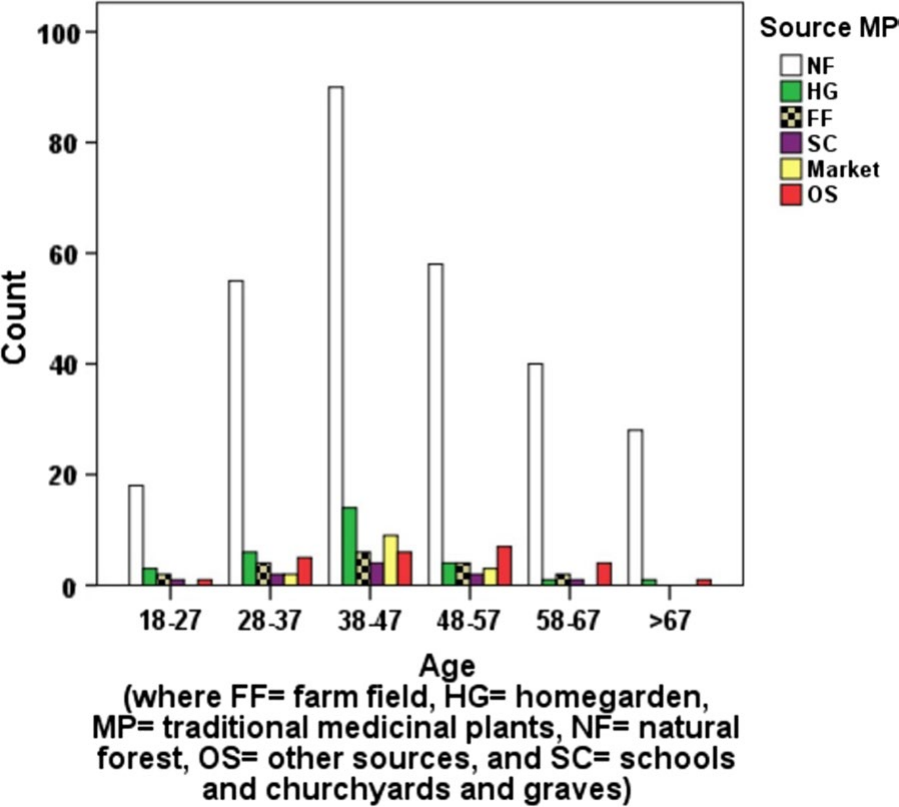
Distribution of age of traditional healers and source of traditional medicinal plants

Most of the traditional healers (230, 59.90%) live nearby and use the natural forest. That is, about 75% of the traditional healers collect medicinal plants from natural forests, but others collect from different sources: farm field, homegarden, and other sources including market (Fig. 2).

**Fig. 3:**
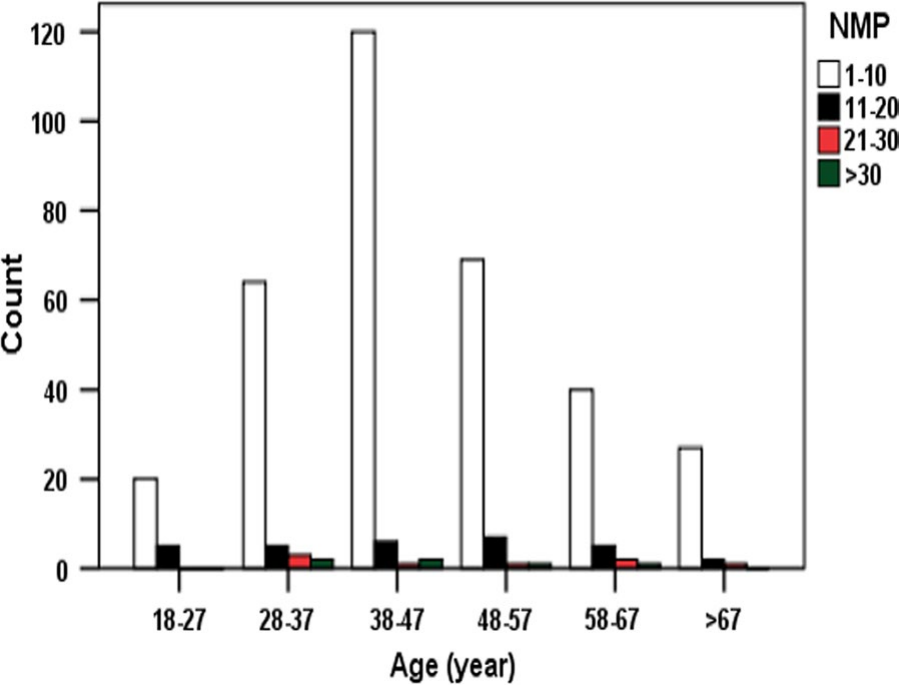
Distribution of age of traditional healers and number of traditional medicinal plants used (NMP)

The number of medicinal plants collected varies (${x}^{2}$=278.368, *df* = 20, *P* = 0.000) with distance from the natural forests at *P* = 0.05 level (Fig. 3).

About 39 TMPs were observed within the local markets. Of these, species sold for medicinal purposes were only six including *Echinops kebericho, Eucalyptus globulus, Gossypium hirsutum, Hydnora abyssinica, Pentas schimperiana*, and *Vepris daniellii.* The other 33 species were sold in the name of spices, condiments, and medicinal (Additional file 3).

#### Knowledge of dosage form and side effects

Traditional healers of the study area know the effects of overdose of the herbal preparations on the health of patients. They provide differential usage information to their patients and determine the dosage based on sex, age, strength of the patient, and nature of the ailment. They prescribe to their patients the days, times, and quantities of remedies to use and provide selected medicine with precautions and timely consultations. They administer through oral route (solid or liquid) or nasal, apply on the ear or eye (drop, ointment, gel), or paste the preparation on the skin; take steam bath, inhale through the nostril, or take a smoke bath.

Of the TMPs, 18 were recorded with some side effects (Additional file 4). Traditional healers confidently know that the side effects like sleeping, dysentery (fluid flatulence), vomiting, bad smell, bloating, and farting, sneezing, and eye redness may occasionally happen after intake of the herbal remedy. For example, like in the liver case, in order to cope up with these they use different techniques. They practice on the issue of coping up and reducing risks in different ways including by advising patients to take rest; drink juice or fresh milk; serve during the early morning hours before breakfast; otherwise decide to mix with ingredients and solvents (food, coffee, water, salt, milk, butter, oil, spices, sugar, honey); not given for pregnant women, children, and the aged as well as people with low or compromised immunity and when they encounter such cases, they refer the patient to the modern health system.

#### Traditional medication as culture

The study area people know that the food they take in is considered medicine. Diversifying the dining table and homestead are the two important cultural norms within the community that helped them to protect themselves from many diseases and famine, environmental stress and to conserve plant biodiversity. There is a saying to assure this “issibay baynnawe gutuma miiki ge”—meaning “nothing at hand but committed not to eat a single food item.” Households with diversified agroforestry, vegetables, enset, coffee, and fresh plants to reap rather than simply eating the single staple foods and for income generation is respected among the community. Even during the wedding process to sustain the planned members of the family in several aspects, it is normally asked as a precondition. To diversify their dining table they collect from different sources like markets and grow a lot of spice and condiment plants, intercropping, and agroforestry in their homegardens for immediate access. So, they developed a culture of diversifying diet by different vegetables, fruits, and other fresh plant materials including spices and condiments, because bringing single food to the table without other diversifying items is a taboo. Just in case that lady was scolded by saying “boozato” meaning “a lazy girl/woman.” She has got to come up with boiled coffee leaf, milk, condiments, and other diversified food sources to assist the convenience of eating the staple food. In these ways, the people encourage ingesting the medicines with the food they eat and serve to others.

In the study area, two forms of traditional medicine were practiced: secret and public. The secret way is practiced by special knowledgeable healers, while the public way is practiced by the general public or laymen of the community. They are aware of the preparation of medications in the kinds of sauces (like Kuubuwa, wos’iya), soups (like Eretsa/C'aba), syrup (juice), and drinks like “haytsa-bunaa”—boiled coffee leaf as supplementary material taken while eating the staple food.

There is special preparation of herbs of plants mainly using coffee leaf as basic component which is locally called as “haytsa bunaa” mainly for respiratory diseases, rheumatism, arthritis, for nursing mother, and sometimes as a part of diet if there is no other condiment and spice or other items. Traditionally, the people also prepare lots of sauces (Kuubuwa), a type of sauce prepared mainly from garlic, cress, mustard, long pepper, pepper chilies, kebercho with cheese and butter, occasionally eaten every morning with local bread for medicinal purpose, especially during the transitional months of rain and dry seasons (October and May).

### Threats of traditional medicinal plants and associated community knowledge

#### Depending on natural forests for treating ailment and other uses

Multipurpose nature and overutilization of TMPs leads to the loss of the plant**.** About 246 (89.79%) species were recorded with one to nine uses, while 28 (10.21%) species were with 10 to 20 uses which indicated their extensive utility. Almost all of the traditional healers claim this as it was confirmed from the use value report (Table 3). As an example, *Croton macrostachyus* recorded with 19 medicinal and 13 other uses; *Syzygium guineense subsp. guineense* with 13 medicinal and 20 other uses*; Vernonia theophrastifolia* with 12 medicinal and 10 other uses, and *Maesa lanceolata* with 8 medicinal and 15 other uses (Table 3).

**Table 3:** Report of some selected TMPs with high use value in the study area

Scientific name	UR	DT	DC	OU	IC %	UV	Rank
*Echinops kebericho*	346	13	7	2	90.1	0.9	1
*Syzygium guineense*	332	13	8	20	86.46	0.87	2
*Pentas schimperiana*	322	11	8	7	83.85	0.84	3
*Maerua oblongifolia*	290	17	7	6	75.52	0.76	4
*Ensete ventricosum*	216	10	8	10	56.25	0.56	5
*Allium sativum*	300	10	7	3	78.13	0.78	6
*Dichondra repens*	220	8	4	3	57.29	0.57	7
*Commelina benghalensis*	210	4	3	4	54.69	0.55	8
*Datura stramonium*	212	7	5	2	55.21	0.55	8
*Cyperus articulatus*	198	3	2	2	51.56	0.52	9
*Centella asiatiaca*	190	9	4	2	49.48	0.5	10

Where UR = use reports, DT = no. of ailments treated, DC = no. of disease clusters, OU = other uses, IC = informant consensus, UV = use value

The results of the relative importance (RI) were computed. The results showed that the most important species among those compared the top ones listed their order of importance the top were *Syzygium guineense, Ensete ventricosum, and Rumex nepalensis* (Table 4).

**Table 4:** Selected TMPs with high relative importance (RI) in the study area

Scientific name	UR	UV	DT	DC	OU	IC %	RI	Rank
*Syzygium guineense*	332	0.87	13	8	20	86.46	2	1
*Ensete ventricosum*	216	0.56	10	8	10	56.25	1.61	2
*Rumex nepalensis*	19	0.05	16	8	3	4.95	1.58	3
*Maerua oblongifolia*	290	0.76	17	7	6	75.52	1.57	4
*Pentas schimperiana*	322	0.84	11	8	7	83.85	1.55	5
*Phytolacca dodecandra*	31	0.08	12	8	4	8.07	1.48	6
*Vernonia amygdalina*	11	0.03	11	6	13	2.86	1.48	7
*Maesa lanceolata*	21	0.06	8	6	15	5.47	1.45	8
*Rossmarinus officinalis*	8	0.02	12	8	2	2.08	1.42	9
*Eucalyptus globules*	79	0.21	9	7	7	20.57	1.36	10
*Juniperus procera*	6	0.02	8	6	12	1.56	1.36	11

Where UR = use report, UV = use value, DT = no. of diseases treated, DC = disease cluster, OU = other uses rather than medicinal value, IC = informant consensus

The computation of use diversity confirmed that there were highly diverse uses (*H* = 5.4656) in the study area. There were important plants with large number of 166 uses recorded. *Croton macrostachyus* (32)*, Vernonia amygdalina* (24), and *Maerua oblongifolia* (23) are the species that came up with highly diversified uses (Table 5).

**Table 5:** Use diversity value of medicinal plants in the study area

Scientific name	UR	DT	UCl	OU	n	(N)	p c	p c2	ln p c	p c ln p c	UDR
*Croton macrostachyus*	144	19	2	13	32	2306	0.0139	0.000193	− 4.277	− 0.059	1
*Vernonia amygdalina*	11	11	6	13	24	2306	0.0104	0.000108	− 4.565	− 0.048	2
*Maerua oblongifolia*	290	17	7	6	23	2306	0.0100	9.95E−05	− 4.608	− 0.046	3
*Maesa lanceolata*	21	8	6	15	23	2306	0.0100	9.95E−05	− 4.608	− 0.046	4
*Prunus africana*	5	4	3	18	22	2306	0.0095	9.1E−05	− 4.652	− 0.044	5
*Vernonia theophrastifolia*	10	12	5	10	22	2306	0.0095	9.1E−05	− 4.652	− 0.044	6
*Schefflera abyssinica*	6	3	2	18	21	2306	0.0091	8.29E−05	− 4.698	− 0.043	7
*Juniperus procera*	6	8	6	12	20	2306	0.0087	7.52E−05	− 4.748	− 0.041	8
*Ensete ventricosum*	216	10	8	10	20	2306	0.0087	7.52E−05	− 4.748	− 0.041	9
*Polyscias fulva*	4	4	2	16	20	2306	0.0087	7.52E−05	− 4.748	− 0.041	10

UR = use reports; DT = number of diseases treated; UCl = number of disease clusters; OU = other uses; n = total number of uses; N = Σn or (UC); pc = n/N; In = natural logarithm; UV = use value; UDR = use diversity rank

Cultural value also showed that the presence of diverse uses is important in the study area. *Syzygium guineense, Maerua oblongifolia*, and *Pentas schimperiana* were with high cultural value 0.15, 0.079, and 0.076, respectively (Table 6).

**Table 6:** Cultural value in the study area

Scientific name	UR	TR	UCL	DT	OU	n	UT	Uce	Ic	IUc	CV_*e*_	Rank
*Syzygium guineense*	332	384	8	13	20	33	166	0.2	0.86	0.86	0.1486	1
*Maerua oblongifolia*	290	384	7	17	6	23	166	0.14	0.76	0.76	0.079	2
*Pentas schimperiana*	322	384	8	11	7	18	166	0.11	0.84	0.84	0.0763	3
*Echinops kebericho*	346	384	7	13	2	15	166	0.09	0.9	0.9	0.0734	4
*Allium sativum*	300	384	7	10	3	13	166	0.08	0.78	0.78	0.0478	5
*Ensete ventricosum*	216	384	8	10	10	20	166	0.12	0.56	0.56	0.0381	6
*Croton macrostachyus*	144	384	2	19	13	32	166	0.19	0.38	0.38	0.0271	7
*Dichondra repens*	220	384	4	8	3	11	166	0.07	0.57	0.57	0.0218	8
*Datura stramonium*	212	384	5	7	2	9	166	0.05	0.55	0.55	0.0165	9
*Centella asiatiaca*	190	384	4	9	2	11	166	0.07	0.49	0.49	0.0162	10

Where UR = use reports; DT = no. of diseases treated; UCl = no. of use clusters; OU = other uses; n = general use cited (UC); UT = use total; CVe = cultural value; Uce = the total number of uses reported for species *e* divided by the total number of potential uses; Ic = the number of participants who listed species *e* as useful divided by the total number of people participating in free listing; IUc = the number of participants who mentioned each use of species *e* divided by the total number of participants

The result of the study showed that the majority of 176 (64.23%) TMPs were rarely distributed in natural forests, while 98 (35.77%) of the species were abundantly distributed in farm fields, homegardens, and natural forests. Irrespective of the distance of traditional healers’ homes from the marketplace, the majority (75.3%) of the healers rely on natural forests (*x*^2^ = 29.73, *df* = 10, *P* = 001) for sourcing the medicinal plants.

The data matrix of the results of ten informants for ten multipurpose traditional medicinal plant species against 12 use categories was computed and indicated that *Ficus vasta, Syzygium guineense subsp. guineense*, and *Maesa lanceolata* are the top threatened species due to their multipurpose nature within the study area (Table 7).

**Table 7:** Results of ten informants for ten multipurpose medicinal plants species

Medicinal plant	Use categories												T	OU	MU	R
	AF	AT	CH	CL	CN	FH	FU	FW	Id	L	Md	SD				
*Croton macrostachyus*	2	4	3	2	5	0	5	5	1	3	5	3	40	13	19	8
*Ficus vasta*	5	5	5	5	5	2	5	5	5	4	5	5	57	13	3	1
*Vepris daniellii*	3	4	0	2	5	4	3	5	0	4	5	3	39	13	4	9
*S.guineense*	2	5	5	5	5	2	4	5	1	4	5	5	54	20	13	2
*Vernonia theophrastifolia*	2	3	0	3	4	0	3	5	5	3	5	1	34	10	12	10
*Juniperus procera*	0	5	0	5	5	0	5	5	3	5	4	5	42	12	8	6
*Prunus africana*	0	5	4	5	5	0	5	5	4	5	5	5	42	18	4	6
*Maesa lanceolata*	3	5	5	4	5	0	5	5	1	5	5	2	50	15	8	3
*Polyscias fulva*	4	5	2	5	5	0	5	5	3	5	5	5	45	16	4	5
*Dombeya torrida*	5	5	4	5	5	0	5	5	1	5	5	1	49	11	2	4
Total	26	48	26	41	49	10	46	50	28	44	49	36	46			

5 = best, 4 = very good, 3 = good, 2 = less used, 1 = least used and ,0 = no value) (where AF = animal feed, agricultural tool, CH = charcoal, CN = construction material, FH = food, FU = furniture, FW = firewood, MU = general medicinal uses, OU = general other uses, I = indicator plant, L = live fence, Md = medicinal, R = rank, SD = shade, T = total

#### Distance from urbanization and modern infrastructures

Most of the traditional healers (190, 49.48%) live near the health center less than 2 km: 146 (38.02%) in between 2 and 4 km, while the rest 48 (12.5%) live far (> 4 km) from the health center. Traditional healing practice has a strong positive correlation with distance from a health center (DHC). As the distance of the residence of healers from DHC and DM increased, the practice of knowledge using large number of medicinal plants (*r* = 0.170, *α* = 0.05, *P* = 0.001) and treating of large number of diseases (*r* = 0.184, *α* = 0.05, *P* = 0.000) was also increased. The usual adage is that traditional medicine lacks information about dose and dosages, expiry dates, prepared under unhygienic conditions; traditional healers were illiterates and practiced in secrecy and spiritual involvement and finally no documentation of the safety were considered as one of threatening factors for the continuance of the healing system itself.

#### The manner of transfer of knowledge and documentation

The death of knowledgeable elder persons before documentation and transferring of indigenous knowledge causes great danger to the plant population, the associated community knowledge, and therefore the service rendered. About 49 (12.76%) respondents were females whose age lies in between 20 and 75 years, while 335 (87.24%) were male respondents recorded with 18 to 92 years of age. The early aged knowledgeable persons are few in number, and over 50% of the traditional healers have traditional healing practice of 11–30 years of experience and also the age lies between 38 and 47 years. The age has direct correlation (*x*^2^ = 19.229, *df* = 10, *P* = 0.037) with transfer of knowledge and (*r* = 0.110, *α* = 0.05, *P* = 0.0432) with documentation. Younger members were willing to transfer the knowledge at an early age but aged ones at their last life span. The way of the transfer of information of plant remedies is one among the threats. As family size increases, the willingness of the transfer of knowledge decreases. It was recorded that the majority of the traditional healers (232, 60.42%) attempt to transfer their knowledge orally to their elder sons with a robust oath at the top of their life span. This was realized in computation that family size has statistically significant relationship (*r* = − 0.126, *α* = 0.05, *P* = 0.014) with transfer of knowledge, because they convey to only their elder son, or elder daughter. Others decided to transfer for any interested individuals.

It has been recorded that about 332 (86.46%) of the traditional healers do not document their knowledge, but only 52 (13.54%) document their knowledge. Most of the healers (208, 54.17%) did not attended grade levels, and education has highly significant correlation (${x}^{2}$= 72.076, *df* = 4, *P* = 0.000) with documentation at *P* = 0.05 level.

#### Service charge for traditional herbal medication

**Fig. 4:**
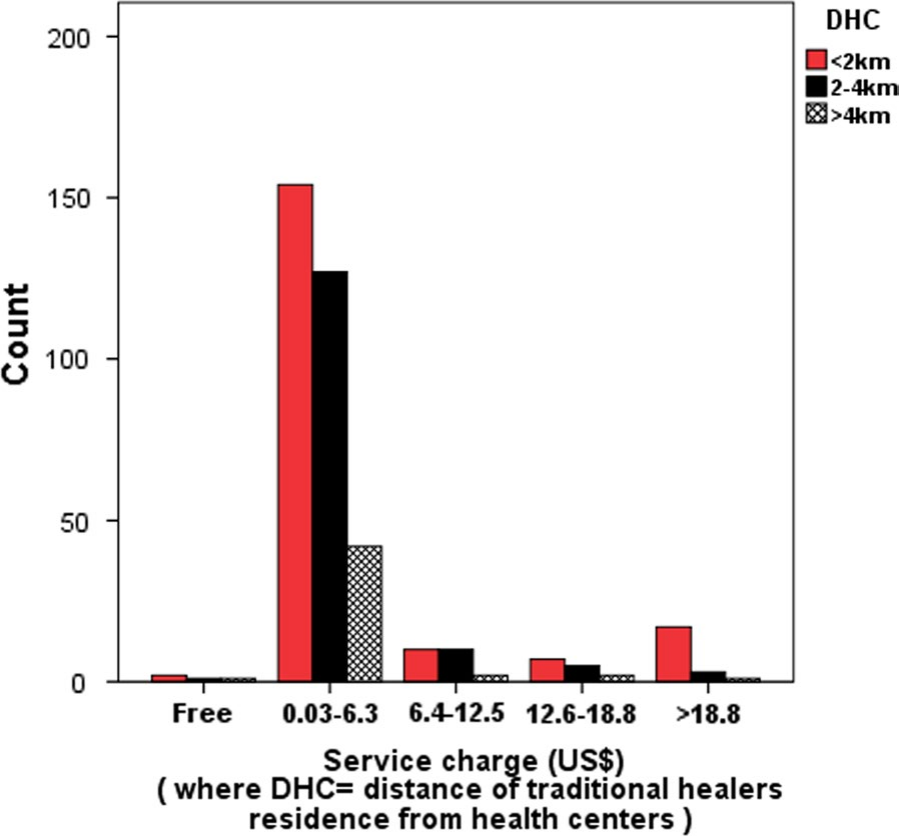
Effect of distance of traditional healers from health centers (DHC) on service charge

Culture allows determining the amount of money or kind to receive for the service they deliver. A good number of traditional healers (182, 47.4%) serve their community by giving the service free of charge, either receive the small amount of cash 0.0313–6.26 US$ (1–200 ETB) for the service they delivered, or otherwise they take a chunk of material like pieces of cloth instead of money (Fig. 4).

There is a positive association of service charge (*x*^2^ = 24.349, *df* = 5, *P* = 0.000) with age, (*x*^2^ = 309.119, *df* = 184, *P* = 0.000) with educational level, and (*x*^2^ = 851.230, *df* = 598, *P* = 0.000) with distance of traditional healer's residence from the health center which was shown to be statistically significant.

## Discussion

### Diversity of medicinal plants within the study area

The THs of the study area use many TMPs, especially species of the Asteraceae and many other families (81) to cure various human and livestock ailments. In contrast, the studies in southern Ethiopia [40, 41] indicated Lamiaceae, in Mideast and Midwest of the country Ethiopia [42, 43] is Fabaceae and in Southeast [44] Solanaceae was the most frequently used family of TMPs.

THs showed preference for herbs to other growth forms of traditional medicinal plants. Similar observations were reported from other areas [40, 41, 43–46] except in the mid-eastern parts of the country [42] which is tree. Their general habitation around natural forests and the availability of herbs throughout the study area and their ease of collection may have been the main reasons for this. Leaf is the most frequently used, and the root is the next TMPs part for treating ailments similar to other areas [40–42, 44, 45] except in the mid-western parts of the country [43] where root and then the leaf comes next. The leaves were preferred for preparation of TM because of the constituency of bioactive compounds, easily accessible and mostly using the food as medicine. The result showed the identical trend elsewhere [13, 47]. The use of leaves sustains the plant than harvesting of the roots. Root use needs mechanisms to shift to the use of other parts of the plant, otherwise implements sustainable utilization modes to reduce the risk of loss. A study undertaken in Kelala [46] and Mana Angetu [48] had also reported the abusive harvesting of roots.

Informants reported that most TMPs are used in decoction to dissolve the chemicals of the material and that they extract its constituents using water and local alcohol (*Harak’iya*). This study agrees with the study in Tigray, northern Ethiopia [49], which reported the use of locally made drinking alcohol for extraction. They use honey to shun the uncomfortable taste of the preparation. This report agrees with the study reported from India [50].

### Ethnobotanical knowledge of medicinal plants

#### Acquisition of traditional medicinal knowledge

Year of healing practice and age of TH are related. The age and stay within the area have positive associations. They should practice with at least the general public traditional medicinal knowledge. Similar was reported to Kenya [19]. This is because of the formation of social intimacy, marriage, and relativity that increases repeatedly visiting community members and chance of sharing skills and knowledge as well as outreaching of knowledge about him/her in the area.

Most of the THs get hold of their awareness from their families in words with great privacy usually at adulthood. The report of west Showa in Ethiopia [51] indicated a similar norm. This is because to keep secrecy, the retaining of knowledge and practice in the family root is needed, the actual training takes long time exercise, and the active and trusted juvenile to keep secrecy and oath is distinguished. Some acquire following shape and color resemblance of nature (the plant) for treatment like in *Tragia cinerea* and *Asparagus racemosus* (climbing behavior) and *Abrus precatorius* (its climbing behavior and color of seed is observed), *Cynodon dactylon, Dichondra repens, and Indigofera spicata* (runner), *Plantago lanceolata* (apical shoot resemblance), *Polyscias fulva* and *Dracaena steudneri* (straight stem with top branches) used for snakebite, *Cuscuta kilimanjari* (its climbing and running behavior and color of stem is observed) for ascaris*, Ensete ventricosum* (red variety color is observed) for retained placenta, and *Gomphocarpus physocarpus* (its hairy balls were observed) for swelling of gland /Lymph adenitis. But, unless the juvenile person who receives the knowledge is interested, documented the event and has a lively memory of understanding and knows the said plants of the realm, there is a leak of information.

Most of the time disease occurred within the study area was human suffering diseases. The study results indicated that TH developed knowledge and filled the gap of the fashionable healthcare system through treating the identical pathological state by using many traditional plants like just in case of liver problems and diarrhea. The identical was reported for south central Ethiopia [52]. The reasons are as follows: 1. Most of them embark in inaccessible areas where there is scarcity of medical institutions, far away from these infrastructures. 2. The traditional medicinal knowledge determines the extent of the employment of medicinal plants for medicinal purposes. 3. Because the burden and practice is exercised in a given area for a very long time, it becomes the culture or trend of the community. TH with knowledge of many medicinal plants has positive association with family size. This is because they need to increase information on how to take health care of their family members with least expenditures and struggle to sustain their relations in several aspects. The similar observation was reported to Wolaita of Ethiopia [53]. Because the number of medicinal plant’s knowledge increases, the amount of diseases practiced to treat increases. TMPs used for the identical disease are more likely to be biologically active and need for phytochemical and antibacterial study. The same study was reported in west Showa of Ethiopia [51]. They claim that curing diseases which were scientifically proven to be incurable like cancers became curable by traditional healing practice of the study area. The identical was reported to the Pinyin society [54].

#### The TMPs used and their sources

Most THs reported collecting their TMPs from natural forests. They also collect them from different sources like markets, institutions (churchyards and graves and schools, farmers’ training centers), rangelands, bushes, marshy areas, and sides of roads. As age increases, both the source and number of TMPs were increased to a certain middle age. However, it decreases beyond 60 years. This is because elders are tired of going to distant places and hence tend to grow them in their homestead and practice their children on their knowledge.

The women, children, and the poor were selling fresh edible and medicinal plants in the market which is different from the report of the culture of Guji in Ethiopia [55] that strictly forbade selling TMP in the market. The sellers within the local market were not TH but local traders and vendors of various materials like spices. This report was similar to the report of Bench and elsewhere in Ethiopia [56, 57]. There is no purposeful marketing of TMPs in the open market. To monitor the practice of traditional medicine needs the legalized system, special spaces, and procedures. This study is different from the report for western Kenya [21].

#### Knowledge of dosage form and side effects

THs of the study area know the effect of overdose use of some TMPs on the health of patients. This study result is different from the report of most communities of Africa [58] that indicates no regulated use and adverse effect [59]. They provide differential usage information and prescription to their patients and determine the dosage supporting different conditions, status of patients, and nature of the ailment with precautions. Otherwise, they refer their patients to the best practitioners or to the modern health system before they deliver the service. This study agrees with the study of Misha Woreda in Hadiya Zone [60] and works reported from India [37] and the Pinyin society of China [54]. This is a practice that they developed a very long time ago and the frequent practice for increasing frequency of customer visitation, not to lose their customers, to point out their ability of identifying the particular disease of the patient and being open to the modernized system of medication in traditional healing practice. The same report from a study undertaken in western Kenya [21] indicated that consumers usually choose traditional medicine because they share common traditional culture, beliefs, relationships, social life, and environment with the traditional medicine practitioners.

#### Traditional medication as culture

Cultural norms of diversifying the dining table and homestead and special preparations of traditional public medication within the community helped them to guard themselves from many diseases and famine, environmental stress and to conserve multipurpose plants. Reports from elsewhere [61–63] also support this norm. This can be because the management designed and practiced by early elders went through centuries of practice. The practice within the community became trendy and developed to the culture that enforces the community members to implement at household level.

### Threats of TMPs and associated community knowledge

#### Dependence on natural forests for treating ailment and other uses

Healers mostly use multiple plants or multiple plant parts to treat one health problem in order to extend the strength and efficacy of the drug. The plant secures a high UD, CV, UV, and RI score that indicates there are many use reports for that plant, while a low score indicates fewer use reports cited by the traditional healers. There were highly diverse uses (*H* = 5.465619632) in the study area. There was a similar report to the Sheka Zone which is 5:155 [64]. This is because both of them were from Southwestern Ethiopia and surrounded by natural forests which increased the interaction to the people living around and encroached. The species were recorded with 2 to 32 uses, but the study to the Bolivian Amazon reported it from one to more than three [36]. This also indicates that the species were the most preferred [37]. This might be due to easily accessible, easily harvested and have high familiarized utilization.

Comparison of the plants said to be used by the people in different parts of Ethiopia with those of the study area showed some interesting observations. The lists of medicinal plants reported in previously published scientific articles were compared with the results of the present study. Of the recorded 274 TMP species of the present study area, 166 had common uses, while 108 were recorded peculiarly in the study area (Table 8; Additional file 5). The reports to Hawassa Zuria of Ethiopia recorded five endemic medicinal plant species [41], but in the present study, there were 11 endemic traditional medicinal plant species to Ethiopia documented including *Aloe otallensis, Coccinia abyssinica, Echinops kebericho, Eragrostis tef, Erythrina brucei, Lippia adoensis, Pycnostachys abyssinica, Satureja paradoxa, Satureja punctata, Trifolium decorum,* and *Vepris daniellii*, confirmed from list of endemic plants of Ethiopia and Eritrea [65].

**Table 8:** Presence and number of common TMP species in different locations and cultures shared with Dawuro communities as counted from some articles published during 2007–2022

No.	Publications	YP	LS	SR	DS	CS	CS %	Rank
1	Wondimu et al.	2007 [42]	Mid-eastern	83	56	27	32.5	12
2	Flatie1 et al.	2009 [43]	Mid-western	40	31	9	22.5	13
3	Mesfin et al.	2014 [44]	Southeastern	56	22	34	60.7	2
4	Bilal et al.	2017 [45]	Eastern	45	22	23	51	4
5	Dirgo	2019 [40]	Southern	102	51	51	50	6
6	Tefera and Kim	2019 [41]	Southern	105	51	54	51	4
7	Teka et al.	2020 [52]	Central	244	153	91	37.3	10
8	Kasaa et al.	2020 [64]	Southwestern	266	159	107	40.2	9
9	Osman	2020 [66]	Northern	91	41	50	55	3
10	Tahir et al.	2021 [67]	Northern	127	81	46	36	11
11	Gebre and Chinthapalli	2021 [68]	Southern	53	18	35	66	1
12	Alemneh	2021 [69]	Northwestern	112	62	50	44.6	8
13	Megersa and Tamrat	2022 [49]	Northwestern	75	41	34	45.3	7

Where CS = common species; DS = no. of different species; LS = location of study in Ethiopia; SR = no. of species reported; YP = year of publication

Apart from the 108 peculiarly recorded traditional medicinal plant species in the study area, the uniquely recorded species with their use were novel to this study. Some of them were *Carduus nyassanus, Dichondra repens, Dissotis canescens*, *Entada abyssinica*, *Euphorbia hirta*, *Hydnora abyssinica, Indigofera arrecta, Launaea capitata*, *Pittosporum abyssinicum, Plectranthus ornatus, Solanum capsicoides*, *Spilanthes mauritiana*, and *Vernonia theophrastifolia.*

Ethnobotanically, *Carduus nyassanus* has novelty of use for blackleg, liver problems, wound healing, scabies, snakebite and anaphylactic shock (devil sickness/evil spirit). *Dichondra repens* is used for headache, wound healing, pyoderma, tinea capitis (ringworm), snakebite (any) and venom, swelling of nymph gland, and bat droppings and its urine. *Dissotis canescens* is used for insect venom, liver problems, snakebite (Goosiya), wound, and evil eye. *Entada abyssinica* is used for snakebite (any), wound, cancer, onchocerciasis, impetigo, liver problems, fever, allergic case, and eye disease. *Euphorbia hirta* is used for liver problems, ringworm, impetigo, snakebite, and wound healing. *Hydnora abyssinica* is used for colic pain, rheumatism, stomachache, body swelling, and wound healing. *Indigofera arrecta* is used for stomachache, colic pain, blackleg, and swelling of gland. *Launaea capitata* is used for stomachache, anaphylactic shock, colic pain, liver problem, tinea capitis (ringworm), snakebite, wound, and evil eye. *Pittosporum abyssinicum* is used peculiarly for intestinal problems, internal parasites, urine problems, diarrhea, swelling of gland, ascaris, diarrhea, and vomiting no elsewhere reported for use. *Plectranthus ornatus* is used for rheumatism, stomachache, internal parasites, and gastritis. *Solanum capsicoides* is used for cough, common cold, rheumatism, fever, allergic case, eye disease, and toothache. *Spilanthes mauritiana* is used for stomachache, evil eye, tonsillitis, animal fattening, milk yield, and dust removal from the eye. *Vernonia theophrastifolia* is used uniquely for anaphylactic shock (devil sickness/evil spirit), eye, wound healing, onchocerciasis, impetigo, colic pain, snakebite, and liver problem (hepatitis, cirrhosis, scaring).

In addition to this, the result of comparison of ethnobotanical studies carried out in different times and parts of Ethiopia with this study also indicated the novelty of the use of some traditional medicinal plants. In the study area, *Centella asiatiaca* is used for gum problem, headache, pyoderma, ringworm, snakebite (any) and venom, swelling of lymph gland and bat droppings and urine, but in Sheka [64] it is used for healing only wound/warts. *Clematis hirsuta* is used in the study area peculiarly for liver problem, tinea capitis (ringworm), fecal cases, and used as detergent. It is also used similarly for snakebite/repellent, wound healing (inflammation, cancer), and headache (painful) in Assosa [43], in North Wollo [66] and in Adwa [68]. In the study area, *Commelina africana* is used for rabies but in Sidama [41] for skin diseases. *Commelina benghalensis* is used for diarrhea and tinea capitis (ringworm); in addition to this, it is used for milk yield and animal fattening, but in Sidama [69] it is used for skin diseases (Chirt).

In the study area, *Cordia africana* is used differently for wound healing, onchocerciasis, and hemorrhage/hemorrhoids from other computed areas and similarly to Sheka [64] and North Wollo [66] in using it for skin diseases like impetigo and jaundice. But, still, it is used for malaria, diarrhea, and dental problem in Amaro [44], for stomachache in Sidama [41], for *Mich*’ acute febrile illness and abdominal illness in Adwa [68], and for involuntary urination in bed and fire burn in North Shewa [49].

In the study area, *Cyathula cylindrica* is used uniquely for snakebite, anthrax, blackleg, and eye disease, but it is reported to Sheka [64] using it for skin disorder. *Cymbopogon citratus* is used for blood pressure, colic pain, and rheumatism but for stomachache in North Shewa [49]. In the study area, *Cynodon dactylon* is used peculiarly for wound healing, shingles (herpes zoster), tonsillitis, snakebite (K'arasha), and stomachache but for eye diseases in North Shewa [49]. *Cynoglossum amplifolium* is used for stomachache, dysentery, diarrhea of children but for Mich in Amaro [44] and for cutaneous sclerosis, hypersensitivity, and sun burn in Sheka [64]. *Cynoglossum lanceolatum* is used for stomachache, snakebite, dysentery, diarrhea of children but for eyelids with cutaneous inflammation, hypersensitivity, and sun burn in Sheka [64]. *Embelia schimperi* is used novelty for gonorrhea and liver problems but similarly for intestinal parasites like tapeworm and ascaris as reported to Sheka [64] and West Gojjam [67].

In the study area, *Ensete ventricosum* is used novelty for mouth and foot disease, anthrax, liver problem, blood clotting, and earache, but it is used similarly as it was reported for wound healing (scabies), animal fattening, and mechanical break of bone in Sidama [41, 69], its red variety for retained placenta in Central Ethiopia [52] and in Sidama [41], for diarrhea in Sheka [64] and in Sidama [69]. But, it was reported to be used differently for amoebic dysentery in Amaro [44], for ascariasis, skin rush, and spider bite in Sheka [64], for inducing abortion, strength, and improved immune function in Sidama [69]. *Eragrostis tef* is used as a novelty for anemia and appetizer, but it is used as antidote for the snake's venom in Sheka [64]. *Erythrina abyssinica* has used novelty for liver problems (hepatitis, cirrhosis, and scarring) and stomachache but differently used for lice, eye disease, arm pain, eye sclerosis, and tonsillitis in Sheka [64].

In the study area, *Euphorbia platyphyllos* has novelty use for liver problems, stomachache, colic pain, skin diseases (like tinea capitis/ringworm), and gland swelling. But its latex is used for retarding poisonous in West Gojjam [67]. *Girardinia diversifolia* is used exceptionally for rabies, diarrhea, and animal fattening but used for tumor/warts in Sheka [64]. *Indigofera spicata* is used for dysentery, diarrhea of children, colic pain, evil eye, and gastritis but as similarly reported to Amaro [44], it is used for snake bite and scabies and to Arba Minch Zuria [40] for stomachache during menstruation and wound healing. *Juniperus procera* is used for body swelling, poison feeding (shooshuwa), snakebite, diarrhea, rheumatism, bedbug, and late placenta, but similarly used for blackleg as it was reported to Arba Minch Zuria [40] and for diarrhea in Sidama [41], and differently used in Arba Minch Zuria [40] for dressing wound (created by Leishmanial), urination problem and bloating, and in North Wollo [66] for Cough. *Kalanchoe petitiana* is used exceptionally for swelling of gland/lymph adenitis, swelling on the mamps (neck area) of ox, toothache, wound healing, dysentery, diarrhea of children, bimple, and liver problems and similarly for eye disease [41], but in different area, it is used for intestinal parasite [40] and stomachache [40, 64], tonsillitis [40, 67], dingetegna and gastritis [41] and for ascariasis, and foot problems (fungal nail, corns and calluses, athlete's foot, plantar warts) [64].

*Leucas martinicensis* has novelty in use for dysentery and diarrhea of children, washing, eye disease, body swelling, uncomfortable condition and as starter material for enset fermentation but differently used for bloating in central Ethiopia [52]. *Lippia adoensis* var *koseret* is peculiarly used for common cold, gastritis, and also used as condiment and spice for milk and butter but it is used for stomachache in Sheka [64]. *Maerua oblongifolia* is peculiarly used for diarrhea, tetanus, meningitis, epilepsy/ anaphylactic shock, gonorrhea, liver problems, rheumatism, stomachache, evil eye, severe abdominal cramp, hookworm, body swelling, mump, eye disease, leech expel, anthrax and for different epidemic diseases but differently used for shivering of cattle in Arba Minch Zuria [40]. *Momordica foetida* is exceptionally used for rabies, stomachache; dysentery of children, abdominal pain (cramp), body swelling, onchocerciasis, impetigo, liver problem, gonorrhea, colic pain, appetizer but similarly used for diarrhea, snake bite and wound [52, 64, 67], and differently used for gastritis and livestock disease [41], evil spirit [52], dyshidrotic eczema, blood clotting, tonsillitis, jaundice, common cold, tetanus, typhoid fever [64]. *Musa x paradisiaca* is used for toothache, blood pressure, and tetanus, but it is used for rough skin in North Wollo [66].

*Nicandra physalodes* is uniquely used for nostril inner problem, appetizer and tonsillitis but it is differently used for leishmania and horse scabies in Amaro [44] and for ectoparasite and brain sharpness in Adwa [68]. *Pavonia urens* is used for colic pain, stomachache, liver problems, diarrhea, and wound healing, but it is used for cattle diseases in Sheka [64]. *Pentas lanceolata* is used for evil eye, liver problems, colic pain, diarrhea, snakebite and retained placenta but it is used only for stomach problems in Sheka [64]. *Pentas schimperiana* is used for internal parasite, eye disease, evil eye, diarrhea, swelling of gland, appetizer, animal fattening and milk yield, and similarly used for constipation (some dehydration) and wound healing like in Arba Minch Zuria [40], mechanical break of bone (bone fracture) like in central Ethiopia [52] but differently used in Sheka [64]for stomach problem and cow disease.

*Piper capense* is used peculiarly for stomachache, rheumatism, colic pain, common cold, liver problem, animal fattening but similarly used for common cold and differently for breast pain in Sheka [64]. *Polyscias fulva* is used for snakebite, wound healing, stomachache and vomiting but it is different for ear pus in Sheka [64]. *Pycnosotachys abyssinica* is used for Allergic case/allergic reaction, eye disease, animal food poison feeding, blackleg, colic pain, evil spirit, and enset wilting, and similarly for stomachache [64] and differently used for bloody urine, typhoid fever, schistosomiasis, musculoskeletal, common cold, bloody diarrhea, headache, and cattle diseases in Sheka [64] and for lumpy skin disease in Sidama [69]. *Rossmarinus officinalis* has novelty use in the study area is used for wound healing, colic pain, impetigo, tinea capitis (ringworm), epilepsy, blood pressure, rheumatism, common cold, diarrhea, and milk teeth and gum problems and similarly used for stomachache as reported to Sheka [64].

*Schefflera abyssinica* is used exceptionally for stomachache, colic pain, and liver problems but in other area it is differently used for animal fattening, endoparasites, gonorrhea and toothache [64] and for snake bite [67]. *Solanum americanum* is used for internal parasites, fever, gastritis, rheumatism, common cold, earache, constipation and colic pain but differently used in other areas for malaria and toothache [41] and snake bite [41, 49].

In study area, *Syzygium guineense* subsp. *guineense* is used for amoeba, diarrhea, colic pain, wound healing, blackleg, stomachache, evil eye, abdominal pain (cramp), liver problem, swelling of gland, kidney infection and leech expel and similarly used for tonsillitis with other areas [64] but differently used in other areas for children diseases (yehitsan beshita) [43], malaria, hemorrhoid, internal worms, snake bite, gonorrhea [44], horse disease [64], and toothache and low milk yield [69]. *Thalictrum rhynchocarpum* is used for insect/worm poison, shingles (herpes zoster), and liver problems but differently used in other areas for stomachache, diarrhea and spider poisoning [64]. *Tragia cinerea* is used for snakebite (all types), eye disease, gland swelling but in other areas for evil eye and wound [52] and anthrax [66]. *Vepris daniellii* is used for rheumatism, colic pain, and animal fattening and similarly used for stomachache and differently for bloody diarrhea in other areas [64]. *Vernonia urticifolia* is used uniquely for diarrhea, vomiting, stomachache, earache, smelly flatulence/farting (bad smell of waste material) and internal parasite but differently used for snake poison in Sheka [64].

The above-documented TMPs have completely novel use in the study area and their pharmacological activity should be further ensured to use at national level, Ethiopia. The findings of comparison shown that people of distinctive regions within the nation utilize the same kind of TMPs for the same sort of maladies. This makes it easier for further efficacy evaluation and drug synthesis from the informed plants. The result of evaluation furthermore indicated that the same kinds of TMPs were utilized for different ailments and/ or different kinds of TMPs were utilized for the same types of ailments. This also informs us the following: 1. The indigenous knowledge has to be collected from different areas of the country from more than 80 ethnic groups especially, from elders (traditional healers) in systematic manner before they leave the court: the same nationally structured interview questions should be prepared and used to explore from each ethnic group by the nearby universities. And, then the database should be formed to serve the people, the world in a wider sense in primary healthcare and tackle current newly emerged diseases and disease resistant microbes that familiarized the present drugs at hand. 2. Capacitating, legalizing, and integrating the service renders in the scarcity of modern health institutions and service providers 3. Filling the gap of the young through awareness creation training and incorporating it into curriculum. 4. The conservation issue of plants should be prioritized urgently, that is interventions of conservation of endangered TMPs to protect the extinction.

Not only traditional medicinal practitioners of the study area but also the traditional medicinal practitioners of the different parts of the country (Ethiopia) are mostly utilizing the same plants with traditional medicinal value. The lists of medicinal plants reported in some published scientific articles were compared with the result of the present study. The top ten common TMP species frequently used for traditional mending purposes in different parts of the country as distinguished based on the publications listed, (Table 9) include *Croton macrostachyus* which appeared in almost all 13 (100%), out of the 13 papers analyzed from literature studies listed as sample in the present study. *Croton macrostachyus* is peculiarly used for colic pain, rabies, inflammation, pyoderma, impetigo, body swelling, allergic case and swelling of midriff of cattle. It is also used similarly with other areas for malaria [40, 44, 64, 67, 69], hemorrhage/hemorrhoids [42], gonorrhea [42, 69], wound healing [40, 44, 64, 69], anthrax [40, 42], blackleg [44, 52], headache [64], eye disease and tapeworm [45, 49], liver problem [45], and ringworm (tinea capitis/tinea corporis) [49]. But it is differently used for gum ailment [42] and stomachache [42, 64], for anti-dot for snake and snake bite [43, 49], scorpion venom, and heart disease [43], for diarrhea [44, 64], for stopping bleeding, swollen parts to shrink, dysentery, trypanosome [40]. For tuberculosis [41, 64], gastritis, goiter [41], sudden illness (dengetegna), indigestion (due to swallowing plastic material)[52], fungal, ascariasis, jaundice [64, 68], tinea versicolor and tinea nigra (fungal) [49, 64], typhoid fever, bone cancer, earache(any) and tumor [64], Tinea nigra [66], spider fatal, yellow fever [68], blood stress, and epitasis (nosebleeds). Some of its other uses include animal feed, bee forage, bee hive, construction, dry fence, enset board, enset pounder, farm tool, firewood, seasonal indicator, shade tree, timber production, and utensils.

**Table 9:** Top ten common TMPs frequently used for traditional healing system in different parts of the country, Ethiopia

Scientific name	[42]	[43]	[44]	[45]	[40]	[41]	[52]	[64]	[66]	[68]	[69]	[67]	[49]	Remark
	ME	MW	SE	E	S	S	C	SW	N	S	NW	NW	N	Total
*Allium sativum*	1	0	1	0	1	1	1	1	1	1	1	1	1	11
*Carissa spinarum*	1	1	1	0	1	1	1	0	1	0	1	1	1	10
*Coffea arabica*	0	0	0	1	1	1	1	1	1	1	1	1	1	10
*Croton macrostachyus*	1	1	1	1	1	1	1	1	1	1	1	1	1	13
*Echinops kebericho*	1	0	1	0	0	1	1	1	1	1	1	1	1	10
*Eucalyptus globules*	1	0	0	0	1	1	1	1	1	1	1	1	1	10
*Olea europaea subsp. Cuspidata*	0	0	1	1	1	0	1	1	1	1	1	1	1	10
*Rhamnus prinoides*	0	0	1	1	1	1	1	1	1	1	0	1	1	10
*Ruta chalepensis*	1	0	1	1	1	1	1	1	1	1	1	1	1	12
*Zingiber officinale*	1	0	1	1	0	1	1	1	1	1	1	1	1	11
	7	2	8	6	8	9	10	9	10	9	9	10	10	107

The location where the studies were done C = central; E = east; M = mid; N = north; S = south; W = west

The traditional medicinal practitioners of the different parts of the country Ethiopia, especially the southern parts, were utilizing the same kind of plants with traditional medicinal value. This is because most of them share the same culture and have centuries of communication of information among themselves. In other words, there were some peculiar TMPs recorded in the study area, because different individuals and ethnic groups have their own knowledge which is different from others.

The result of the data matrix informed, overexploitation of multipurpose traditional medicinal plant species for construction materials, charcoal production, firewood collection, lumbering and for other purposes accelerated depletion of the species within the study area. Both nearby and distant residents of natural forests with a few years of healing practice rely more on natural forests. The extensive use of roots for various purposes also poses threats of depletion of traditional medicinal plants. This suggests the presence of more pressure on resources exposing them to loss. Multipurpose nature of TMPs ends up in overutilization. The loss of multipurpose plants leads to the loss of indigenous knowledge tied with them, resulting in health problems. This needs conservation and sustainable utilization intervention. The same threat was reported from investigations of different parts of the country that the natural forest is the main source, and anthropogenic activities were the main debilitating factors of TMPs [40–46, 48, 49, 52, 64, 66–69]. This informs us to aware people about conservative utilization for sustainability of vascular plants with diverse use.

#### Distance of urbanization and modern infrastructures

The THs claimed their fear of the modern health system and urban lifestyle as threatening factors. The roads, buildings, and other infrastructures were constructed for development activities, urbanization, and resettlement at the expense of TMPs. It also has influence on other imperative plants and allied community information, and culture and lifestyles. This agrees with the report to Jimma in Ethiopia [70]. The computational result showed that traditional healing practice incorporates a strong correlation with distance from a health center and urban areas. The more the traditional healers living near health centers and urban areas, the more threat to his/her traditional medicinal knowledge will occur. In other words, traditional healing practice is more implemented as the distance far away from modern health institutions. The reason for this is as follows: 1. Some people are not ready to travel to a far place because they probably want to save time and energy. 2. Most people of low income cannot afford the price of modern medication; they prefer the cheapness and effectiveness. This agrees with the report to Jimma in Ethiopia [70] and among the Samia of Funyula Division, Busia District, Kenya [19]. This can be because the practice of time-honored remedy within the study area faced lots of challenges since the beginning of modern medication. THs claim that the negative effect of infusion of legal and health law issues, demoralizing sayings and considering as backward remote activity. This enforces the knowledgeable persons to go away from traditional healthcare systems or distant places where not frequently contact with, consequently, to the loss of healing knowledge. This study result agrees with the report elsewhere in Ethiopia [10], reported to Jigjiga of eastern Ethiopia [45], for western Kenya [21], and elsewhere [71, 72]. The traditional medicine practice has to get formal and legal ground as well as integrate with the modern system to serve the community.

#### The manner of transfer of knowledge and documentation

Most of the traditional healers of the study area claimed that they do not document their knowledge, the TMPs they use, the service they render, and following manner of oral acquisition and conveying of their knowledge and plant remedies. This study agrees with a report from western Kenya [21] which constitutes a system of informal and unwritten regulations. The manner of the transfer of knowledge of plant remedies is one of the threats posed as a result of the oral nature of transmitting the knowledge to their elder son with a robust oath at the tip of their lifespan. On the other hand, the training of treatment takes place with burly realistic mechanism over an extended period under strict supervision and guidance of inheritor, usually the father. This study agrees with the study made on the medicinal practices of the Zay people [73] and in most ethnic societies of Ethiopia [74]. This is due to the following: 1. The healers did not attend grade level education, 2. such convey became a fashion within the study area, 3. to keep the secrecy of knowledge, and 4. the belief that makes public causes the mauling, failure of information and inefficacy while practicing it. This fashion may negatively affect its continuity and would likely end up in the loss of valuable information, TMPs, and deprivation from primary health service.

#### Service charge for traditional herbal medication

The higher the service charge, the more the traditional healer is engaged in the service. The amount of charges the THs receive depends on their age, tenure status, educational level, and distance of their residence from health centers, and on the economic status of patients and the number of patients visiting them. The highest quantity of money determined for the service delivered by young, more educated and nearby healers of the health institutions was 46.95 US$. They receive a massive amount of cash compared to people who are elders. This is because they are ready to face the challenges that will come consequently. The patient revisits THs for thanksgiving after getting their health become okay. The same was reported to Cameron [75] and Africa [76] in that THs receive payment after the patient is cured.

Elders, who stayed for many years in the given area, usually requested a small amount of money, cash 0.0313–6.26 US$. This study disagrees with the study for Jimma town of Ethiopia whose income range is 50.1–250.5US$ [70] and Bui Division of Cameroon 5–100 US$ per month [77]. This is because of the formation of social intimacy (marriage and relativity, colleagues) that their year age and their years of stay in the area increase. As the number of family sizes increases, the social relation, intimacy, recognition, respecting, and sympathy to repeatedly visiting community members also increase. This study agrees with similar study results reported to Jigjiga of eastern Ethiopia [45], and the study among the Samia of Funyula Division, Busia District, Kenya [19], revealed that the association of age and illiteracy with high use of traditional medicine.

The study area traditional healers were treating those could not meet the expense of the cost for present medications. They serve their community free of charge; otherwise they take a chunk of material like pieces of cloth because they think that TMPs might not be effective and also the knowledge cannot further work for others. Culture allows determining the amount of money or kind to receive for the service they deliver which is different from the culture of Guji in Ethiopia [54] that does not allow money to compensate. However, the study area THs do not engage in healing service unless an occasional case happens because their primary and official duty is farming. There is no grant for continuation of the practice because of absence of or low income generation. A similar observation of practice was reported in Wolaita [53].

The younger knowledgeable THs were few in number and have traditional healing practice of a small number of year of experience. The young healers claim that it should be regular service for income generation and mastering the procedures and remedies. They are telling that they work because of the oath of their fore parents for practice continuity. Additionally, deep rooting in culture, respect from community members and extensive practice due to simple identification, approaches and communication, accessibility, cheapness, and effectiveness retained the practice of traditional medicine and continued for hundreds of years without support from elsewhere. Similar study result reported to Jigjiga of eastern Ethiopia [45] support this.

In other words, there have been few TH left in adulthood. The young are to detach the loop and the elderly are close to departing, leaving no traditional medicinal knowledge to the following generation. There is a desire to give confidence and capacitate TH to practice and renovate their service to resolve the globe’s unhealthiness. The integrated approach has been widely practiced and accepted as an effective method for reducing the side effects, minimizing toxicity, reinforcing the treatment efficacy, and reverting multidrug resistance as known from studies in China [64] as a lesson. Therefore, allowing working side by side with modern healthcare systems within the community elongates the indigenous knowledge other than filling the gap of contemporary healthcare system scarcity and helps them to use their potential and avoid the trendy threat posed from different directions.

## Conclusion

The study in Dawuro Zone of Ethiopia documented diversified TMPs both in type and use as applied for the health care of the community and their domestic animals. The source of remedies mostly depends on herbs of natural forests and their preparation on leaves; however, the root comes as the next frequently used plant part. This needs further investigation to determine the level of the risk and to design sustainable harvesting and utilization schemes. TMPs used for the identical disease need phytochemical analysis and antibacterial study to isolate for clinical use.

The agricultural activities, urbanization, low or no charge for the healing service, the secrecy and oral transfer of the knowledge, and the highest demand for medicinal and other multiple purposes (multipurpose nature of the species) were some of the threatening factors for the resource and the associated knowledge as well as the service in the study area. Developing conservation intervention and a sustainable system of utilization is needed for multipurpose medicinal plants. Homestead diversifying norm of the community needs to be encouraged and scaled up to hold back the pressure on natural forests.

The current documented information on the TMPs can be used as a baseline data for future studies of pharmacological and phytochemical investigations. Finally, national integrated assessment on indigenous knowledge about the utilization and status of TMPs before the knowledgeable people leave or die, and incorporating the practice in school curricula to raise awareness of the young members of the community is needed. Furthermore, skill upgrading and science-based training programs and other necessary support and empowerment of the knowledgeable and responsible traditional healers is worthwhile for a well-functioning practice and appropriate integration into health systems for achieving universal health coverage and sustainable development.

## Supplementary Information

**Additional file 1**. **Appendix 1.** Ethnobotanical information of 274 traditional medicinal plants collected from 384 traditional healers of Dawuro Zone in southwestern region of Ethiopia.

**Additional file 2**. **Appendix 2.** Types of diseases, their clusters, the ailments used, and the informant’s consensus factor.

**Additional file 3.** **Appendix 3.**. Some traditional medicinal plants sold in the market of Dawuro Zone in Southwest Region of Ethiopia.

**Additional file 4**. **Appendix 4.**. The list of some TMPs known with their side effect in Dawuro Zone of Southwestern Ethiopia.

**Additional file 5**. **Appendix 5.** Comparing TMPs of the study area with different parts of the country, Ethiopia.

## Data Availability

All data collected for this study were analyzed, interpreted, and included in this manuscript, and its supplementary materials were attached as Additional file 1–5, but other datasets used and/or analyzed during the current study are available from the corresponding author on reasonable request.

## Abbreviations

ANOVA: Analysis of variance
CSA: Central Statistical Agency
WHO: World Health Organization
ICF: Informant consensus factor
HH: Household
THs: Traditional healers
TMPs: Traditional medicinal plants
FL: Fidelity level
SciDevNet: Science for Development and Network
